# Characteristics of Environmentally Persistent Free Radicals in PM2.5 and the Influence of Air Pollutants in Shihezi, Northwestern China

**DOI:** 10.3390/toxics10070341

**Published:** 2022-06-21

**Authors:** Feifei He, Jianjiang Lu, Zhuoying Li, Min Li, Zilong Liu, Yanbin Tong

**Affiliations:** Key Laboratory of Environmental Monitoring and Pollutant Control of Xinjiang Bingtuan, School of Chemistry and Chemical Engineering, Shihezi University, Shihezi 832003, China; hffdeemail@163.com (F.H.); lizhuoying_2020@163.com (Z.L.); limin@shzu.edu.cn (M.L.); liuzilong1999@163.com (Z.L.); tongyanbin@sina.com (Y.T.)

**Keywords:** environmentally persistent free radicals, inhalation exposure, polycyclic aromatic hydrocarbons, air pollution

## Abstract

Environmentally persistent free radicals (EPFRs) are a kind of hazardous substance that exist stably in the atmosphere for a long time. EPFRs combined with fine particulate matter (PM_2.5_) can enter the human respiratory tract through respiration, causing oxidative stress and DNA damage, and they are also closely related to lung cancer. In this study, the inhalation risk for EPFRs in PM_2.5_ and factors influencing this risk were assessed using the equivalent number of cigarette tar EPFRs. The daily inhalation exposure for EPFRs in PM_2.5_ was estimated to be equivalent to 0.66–8.40 cigarette tar EPFRs per day. The concentration level and species characteristics were investigated using electron paramagnetic resonance spectroscopy. The concentration of EPFRs in the study ranged from 1.353–4.653 × 10^13^ spins/g, and the types of EPFRs were mainly oxygen- or carbon-centered semiquinone-type radicals. Our study showed that there is a strong correlation between the concentrations of EPFRs and conventional pollutants, except for sulfur dioxide. The major factors influencing EPFR concentration in the atmosphere were temperature and wind speed; the higher the temperature and wind speed, the lower the concentration of EPFRs. The findings of this study provide an important basis for further research on the formation mechanism and health effects of EPFRs.

## 1. Introduction

The exceedingly high levels of air pollution across China pose a significant threat to public health [1]. Despite the improvement in the air quality in recent decades, the range of particulate matter (PM_2.5_) concentrations across China continues to be associated with increased mortality [2]. Most of the negative effects of PM_2.5_ are related to the overly complex composition of PM_2.5_ [3]. Environmentally persistent free radicals (EPFRs), a kind of environmentally harmful substance that is hazardous to the environment and human health, exist widely in atmospheric [4], soil [5], and water [6] environments. Compared to common free radicals, EPFRs are relatively stable organic free radicals with lifetimes varying between months and years. Biotoxicological studies have found that EPFR-containing PM_2.5_ can produce reactive oxygen species throughout the redox cycle, which can induce oxidative stress [7] and DNA damage [4].

In recent years, researchers have begun to pay attention to the prevalence and risk characteristics of EPFRs in the atmospheric environment. Levels and sources of airborne EPFRs have been reported previously in many studies [8,9]. EPFRs can accompany the generation of particulate matter during the combustion of biomass and biofuels [4,10]. In addition, EPFRs can also be derived directly from coal combustion and traffic emissions [11]. In Beijing, the average EPFR concentration in PM_2.5_ is 6.00 × 10^17^ spins/m^3^ on hazy and non-hazy days, and the average daily inhalation of EPFRs in PM_2.5_ was assessed to be equivalent to 33.1 cigarette tar EPFRs [12]. Existing research indicates that the types, sources, and risks of EPFRs vary depending on the region.

In the 1950s, cigarette smoke was found to have a potentially toxic, cancerous effect due to the presence of persistent free radicals [13]. The spectrum of the EPFRs detected in cigarette tar is comparable to that of the EPFRs found in PM_2.5_, and both can cause similar diseases [14]. The g-factor is a spectral splitting factor that is used for the initial discrimination of free radical species, including semiquinone [15], phenoxy [16], and other types of EPFRs. The g values of persistent radicals in atmospheric particulate matter range from 2.0030 to 2.0047 and correspond to oxygen-centered radical species, such as quinone, semiquinones, and methoxybenzenes [17,18]. Quinone radicals are also tar paramagnetic species in cigarettes and have been reported to be directly involved in the carcinogenic properties of tar [12].

The primary sources of EPFRs include incomplete combustion [9] and thermal treatment of fossil fuels [11], such as vehicle exhausts, waste combustion, and coal burning. The secondary sources of EPFRs are derived from the intermediate products formed by the degradation of organic compounds under natural conditions, such as the degradation of aromatic hydrocarbons and their auto-oxidation processes [19]. Polycyclic aromatic hydrocarbons (PAHs), as precursors, can react heterogeneously with ozone to produce oxygenated functional groups [20], which can stabilize on transition metal surfaces to form EPFRs [21]. Researchers have found that the formation of EPFRs may involve many organic chemical degradation processes [19,20,21,22]. Compared with EPFRs formed under extreme conditions, such as during combustion or under high temperature [22], the formation of EPFRs in natural ambient surroundings is common, but few studies have explored this environmental process. The study of the mechanisms of formation and stabilization of EPFRs under natural conditions will provide new perspectives on the environmental fate, as well as the risk assessment, of organic compounds.

Risk assessments are usually based on a toxicity database for a single contaminant, but the toxicities for mixtures of contaminants are unknown. PAHs are a class of persistent organic pollutants with high health risks that are ubiquitous in the environment [23]. In health risk assessments based on the concentrations of such pollutants, past studies only focused on the toxic effects of a single pollutant, and often ignored pollutants such as EPFRs. Therefore, risk assessments are often unable to evaluate the actual environmental health risks caused by exposure to air pollution. Establishing a methodology for the risk assessment of the combined toxicity of two or more pollutants is an important problem that needs to be resolved. This study investigated the potential health risks posed by PAHs and EPFRs in PM_2.5_ using alternative assessment methods, as well as the correlations between EPFRs and PAHs, to determine possible conversion relationships.

## 2. Materials and Methods

### 2.1. Sampling of Ambient PM_2.5_

The sampling site was at Shihezi University (44°18′ N, 86°03′ E) in Xinjiang, northwestern China. The site is about 20 m from the ground and is positioned on top of the laboratory building. There are no tall structures or significant contamination sources in the vicinity of the sampling site, which is primarily a residential area. There is only one street with traffic in the vicinity; therefore, the sampling site was a representative residential area in Shihezi city.

Sampling campaigns were conducted from October 2020 to September 2021 and represented spring, summer, fall, and winter, respectively. A total of 144 samples were obtained. PM_2.5_ was collected for 23 h using a low volume air sampler (manufactured by BGI, San Jose, CA, USA) with a flow rate of 16.7 L/min. The filter membranes were placed in a muffle furnace at 600 °C for 7 h before sampling, with the aim of eliminating effects from the organic matter adsorbed on the membrane surface. The membranes were weighed after being kept at constant humidity and weight for 24 h before and after sampling, and the daily weather information for the temperature, atmospheric pressure, and humidity were recorded at the time of sampling. After the above work was completed, the membranes were stored in filter boxes at −20 °C.

### 2.2. Analysis of EPFRs in PM_2.5_

The quartz fiber filter was cut into strips of 1 to 2 cm and placed in a quartz tube. EPFRs in PM_2.5_ samples were measured using a Bruker A300 Electron Paramagnetic Resonance Spectrometer (GER). The operating settings of the apparatus were set as follows: The modulation frequency and frequency were 100.00 KHz and 9.84 GHz, respectively. The modulation amplitude was 2.00 G. The sweep width was 100.00 G and the time constant was 40.960 ms. The sweep time was 60.7 s and the microwave power was 20.00 mW. Bruker’s Xenon program was employed to determine the isolated absolute spin of the free radicals and to compute the concentration of the free radical spin. The g-factor and linewidth (ΔHp-p) were measured using Bruker’s Win EPR software. All operations described above were performed at indoor temperature.

### 2.3. PAHs Analysis

An accelerated solvent extraction extractor (E-916, BUCHI, CHE) was employed for the chemical extraction of 16 particle-bound PAHs, including naphthalene (Nap), acenaphthylene (Acy), acenaphthene (Ace), fluorene (Flu), phenanthrene (Phe), anthracene (Ant), fluoranthene (Fla), pyrene (Pyr), benzo[a]anthracene (BaA), chrysene (Chr), benzo[b]fluoranthene (BbF), benzo[k] fluoranthene (BkF), benzo[a]pyrene (BaP), indeno [1,2,3-c,d] pyrene (IcdP), dibenz[a,h]anthracene (DahA), and benzo[g,h,i]perylene (BghiP). Half of the quartz filter used was sliced into small pieces and then put in the extraction tank. A ratio of dichloromethane: hexane equal to 3:1 was used as the extractant. Extraction was performed at 100 bar with a hold time of 5 min and two cycles. After sample concentration, we used ultra-performance liquid chromatography (ACQUITYUPLC, Waters, Milford, CT, USA) to quantify and separate particle-bound PAHs. The UPLC column temperature was set to 35 °C, and the detection wavelength of the diode array was 220 nm. The sample injection volume was 10 μL. The R regression equation, R^2^, detection limit, and mean recovery for the 16 PAHs are shown in Table S1. Field blanks were brought to the sampling sites and the same analytical methods were used to determine the background contamination as for the field samples. In all analyses, the PAH concentrations in the blank samples were below the method detection limits.

To ensure sample accuracy, all operational and analytical processes strictly followed quality assurance and quality control procedures.

### 2.4. Degradation of PAHs and Formation of EPFRs under Different Temperatures

Initially, the concentrations of PAHs and EPFRs were measured at room temperature (20 °C). Then, the effects of different temperatures on PAHs and EPFRs were investigated. The temperature gradient was set to −5 °C, 0 °C, 10 °C, 20 °C, 25 °C, 30 °C, 35 °C, and 40 °C according to the actual ambient atmospheric temperature. PAHs and EPFRs can exist stably at low temperatures [24], so no temperatures were chosen below 0 °C, except for −5 °C as the reference value. The filter membrane samples collected for PM_2.5_ were placed in a beaker with the collection sides of the particles facing upward, the beaker was then placed in a constant temperature and humidity chamber, and the concentration values of PAHs and EPFRs were measured after allowing the samples to rest at a specific temperature for 30 min. In this study, the temperature (10–40 °C) variation was controlled by a constant temperature and humidity chamber, and the humidity was controlled at 65–75% RH. The −5 °C and 0 °C samples were placed in a refrigerator under the same humidity level. To avoid effects from light, the above experiments were performed under light-proof conditions.

### 2.5. Human Exposure Risk Assessment for PAHs and EPFRs

Studies have shown that EPFRs can induce various types of cardiac and breathing system disorders in rats [25], analogous to those noted with smoking [26]. To assess the potential health risks for Shihezi residents exposed to PM_2.5_-bound EPFRs, the same number of cigarettes was used to represent the EPFR exposure per person per day. The calculation formula was as follows:

(1) $$N_{\mathrm{cig}}{=(C}_{\mathrm{EPFRs}}{\;\times \;F\times C}_{\mathrm{PM}2.5}{\times \;\mathrm{IR}\;\times \;F}_{r})/{(\mathrm{BW}\;\times \;\mathrm{RC}}_{\mathrm{cig}}{\times \;C}_{\mathrm{tar}})$$

where N_cig_ stands for an equal number of EPFRs in PM_2.5_ and cigarette tar, C_EPFRs_ indicates the average mass concentration of EPFRs (spins/g), and C_PM2.5_ indicates the PM_2.5_ concentration (μg/m^3^). The respiratory rate of an adult is denoted IR (20 m^3^/day), the alveolar part of the pulmonary region is denoted Fr (0.75), and 70 kg represents the weight of an adult, expressed as BW. The concentrations of EPFRs in cigarette tar are expressed as RCcig (9 × 10^16^ spins/g tar) and the average tar yield per cigarette is 0.031 g, represented by C_tar_.

The toxicity of PAHs is generally assessed by the equivalent concentration of benzo(a)pyrene (BaP). The carcinogenic potency of general commercial cigarettes was assessed using BaP-equivalent concentrations in [27], and the mean BaP-equivalent concentration of commercial cigarettes was 71.7 ng/cig. To assess the potential health risks posed by PAHs and EPFRs in PM_2.5_, the same numbers of cigarettes were used to represent the PAH exposure per person per day. The calculation formula was as follows:

(2) $${\mathrm{BaP}}_{\mathrm{PAHs}}=\sum C_{i}{\;\times \;\mathrm{TEF}}_{i}$$

(3) $$P_{\mathrm{cig}}{=(\mathrm{BaP}}_{\mathrm{PAHs}}{\;\times \mathrm{IR}\times \;F}_{R})/{(\mathrm{BW}\;\times \;F}_{i})$$

where BaP_PAHs_ indicates the equivalent concentration of PAHs, C_i_ represents the concentration of the i-th PAH, and TEF_i_ indicates the toxic equivalency factor [28], as shown in Table S2. N_cig_ represents the equivalent number of PAHs in PM_2.5_ and in cigarette tar and Fi represents the equivalent concentration of BaP in a cigarette (71.7 ng/cig).

## 3. Results and Discussion

### 3.1. EPFR Concentration and Seasonal Variation in PM_2.5_

**Figure 1:**
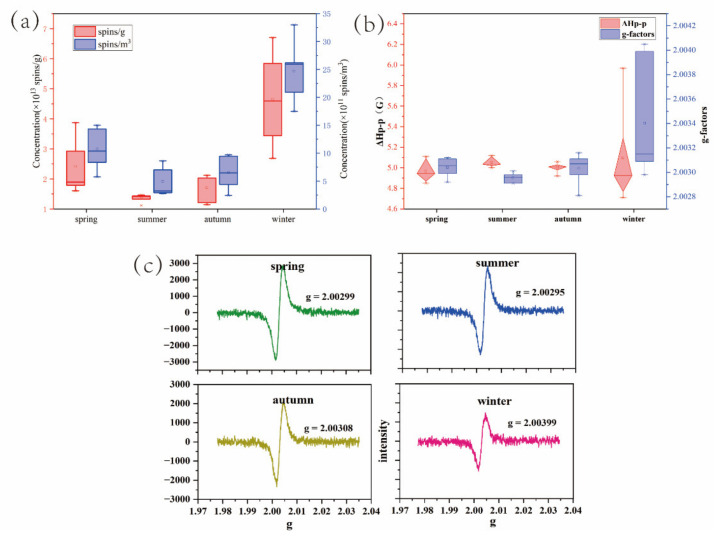
Seasonal distribution trends for EPFR concentrations (**a**), g-factors and ΔHp-p values (**b**), and the EPR spectra (**c**) in PM_2.5_ in Shihezi between October of 2020 and September of 2021.

Figure 1a shows the mean concentrations of EPFRs in PM_2.5_ in spring (2.416 × 10^13^ spins/g), summer (1.353 × 10^13^ spins/g), fall (1.708 × 10^13^ spins/g), and winter (4.653 × 10^13^ spins/g). The comparison of atmospheric EPFR concentrations in different seasons shows that the concentration of EPFRs in winter is remarkably greater than in the other three seasons, which can be attributed to the increase in primary source emissions in winter. The total EPFR concentration increased with the PM_2.5_ concentration, and the highest concentrations of EPFRs and PM_2.5_ appeared in winter. Coal combustion and the use of other fossil fuels, which are contributors to EPFRs, increase dramatically during the winter months. A study conducted by Xu et al. in Beijing, China, in 2018 showed that the average concentration of EPFRs in PM_2.5_ was about 3.75 × 10^21^ spins/g [29]. The average concentration distribution of EPFRs in the air was consistent with that in PM_2.5._ In addition to the seasonal distribution, less rainfall and relatively low temperatures lessen the chances of pollutants diffusing and transforming.

The detection of PM_2.5_ containing EPFRs using electron paramagnetic resonance spectrometry at chamber temperature showed that the mean g-factor was between 2.0028 ± 0.0001 and 2.00405 ± 0.0001 (Figure 1b), indicating that the EPFRs were predominantly oxygen-centered or carbon-centered semiquinone-type radicals. The average ΔHp-p value for the EPFRs tested in PM_2.5_ was 5.03 G (range: 4.71–5.97) in Shihezi. The ΔHp-p values for EPFRs in all four seasons were similar, sugesting that the EPFRs in PM_2.5_ have a similar central carbon structure. However, the chemical constituents can vary considerably depending on the emission source [30]. It should be noted that a larger ΔHp-p value indicates a lack of hyperfine split structures [31], implying the existence of several organic free radicals in PM_2.5_.

### 3.2. Correlation Analysis

**Figure 2:**
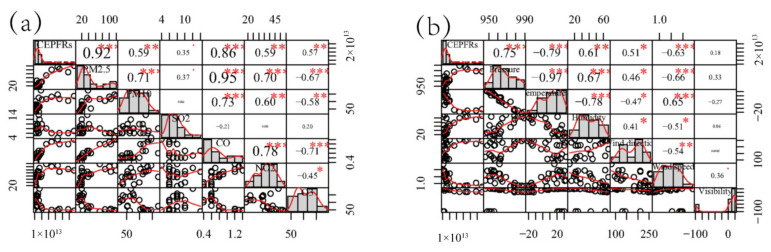
Pearson’s correlation coefficients for atmospheric concentrations of environmentally persistent free radicals and conventional pollutants (**a**) and meteorological variables (**b**).

Correlations between EPFR concentrations, conventional atmospheric pollutants, and meteorological parameters were investigated. Pearson’s correlation analysis was employed to test the linear relationship between factors. There were positive significant correlations between the EPFR concentration and PM_2.5_, PM_10_, CO, NO_2_, and O_3_ (*p* < 0.05), with Pearson’s correlation coefficients of 0.92, 0.59, 0.86, 0.59, and 0.57, respectively (Figure 2a). A weak positive correlation was found between EPFRs and SO_2_. Persistent free radicals can be stabilized in particulate matter, which means that the higher the PM_2.5_/PM_10_ concentration, the more EPFRs there are in the PM_2.5_/PM_10_. The correlation relationship between EPFRs and NO_2_ indicates that coal and petroleum combustion were probably the dominant sources of EPFRs in PM_2.5_ in Shihezi. PAHs can react heterogeneously with ozone to produce a variety of oxygenated functional groups [20]. Therefore, under the condition of high ozone concentration, more free radicals will be generated. The study by Chen et al. (2019) also showed that the concentration of PM_2.5_ combined with EPFRs was significantly positively correlated with PM_2.5_, SO_2_, NO_2_, and O_3_ [11], which is consistent with our findings.

The effects of atmospheric parameters, including pressure, temperature, humidity, wind direction, wind speed, and visibility, on EPFR concentrations were also investigated. A significant negative correlation between temperature and EPFR concentration was observed (*r* = 0.79) (Figure 2b). A previous study [32] showed that temperature affects the properties and concentration of EPFRs. Organic compounds can decompose or react to form free radicals at room temperature [33], and wind promotes the transportation and scattering of EPFRs. This study found a positive correlation between EPFRs and wind direction (*r* = 0.51, *p* < 0.01) and a negtive correlation between EPFRs and wind speed (*r* = −0.63, *p* < 0.01). There were positive significant correlations between EPFR concentration and pressure, humidity, and visibility, with Pearson’s correlation coefficients of 0.75, 0.51, and 0.57, respectively.

### 3.3. Correlation between PAHs and EPFRs

**Figure 3:**
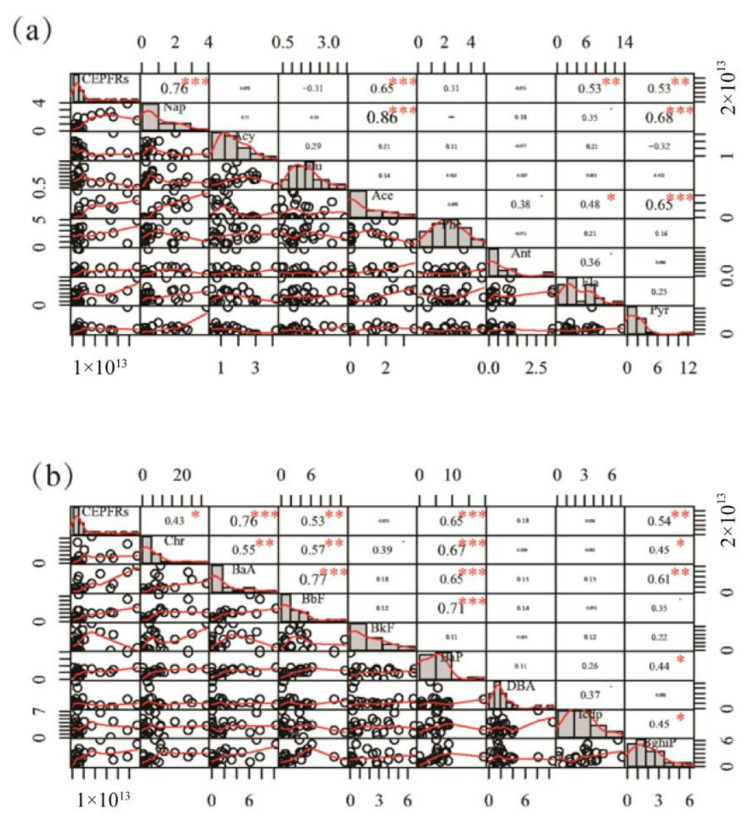
Pearson’s correlation matrices for atmospheric concentrations of environmentally persistent free radicals and eight monomers of PAHs (Nap, Acy, Flu, Ace, Phe, Ant, Fla, and Pyr) (**a**), and a further eight monomers of PAHs (Chr, BaA, BbF, BkF, BaP, DBA, Icdp, and BghiP) (**b**).

The concentration and seasonal variation of 16 PAHs in PM_2.5_ are shown in Figure S1. The correlation between PAHs and EPFRs in PM2.5 was investigated (Figure 3). Due to the conversion between electron gain and loss during the degradation process in organic pollutants, EPFRs with strong oxidative activities can be formed, which are a class of chemical substances with significant environmental risks. PAHs are first adsorbed on particles and, under the action of environmental conditions (such as burning and ultraviolet radiation), chemical changes occur and free radicals are generated [34]. The free radicals interact with inorganic minerals or organic matter, becoming more persistent in the environment and migrating into the environmental medium as aerosols [16]. They then combine with biological processes and, thus, pose a risk to human health. The main behavioral characteristics of PAHs are dependent on the presence of solid particles.

Significant positive correlations (*p* < 0.01) between EPFRs and Nap (0.76), Ace (0.65), Fla (0.53), Pyr (0.53), BaA (0.76), BbF (0.53), BaP (0.65), and Bghip (0.54) were observed, indicating that these organic compounds may have had a major role in the formation of EPFRs in Shihezi. A previous study [35] demonstrated that Pyr and BaA act as representative trace agents of CNG. The high correlation between EPFRs and Pyr and BaA indicates that the source of these EPFRs was natural gas combustion. Nap, Ace, Pyr, and BaA were primarily attributed to coal combustion, indicating that this may be another source of EPFRs. Phe (0.31), Ant (0.073), Chr (0.43), and Icdp (0.064) had weak positive correlations with EPFRs, while weak negative correlations were found between EPFRs and Acy (−0.072), Flu (−0.31), BkF (−0.056), and DBA (−0.18).

### 3.4. Degradation of PAHs and Formation of EPFRs

**Figure 4:**
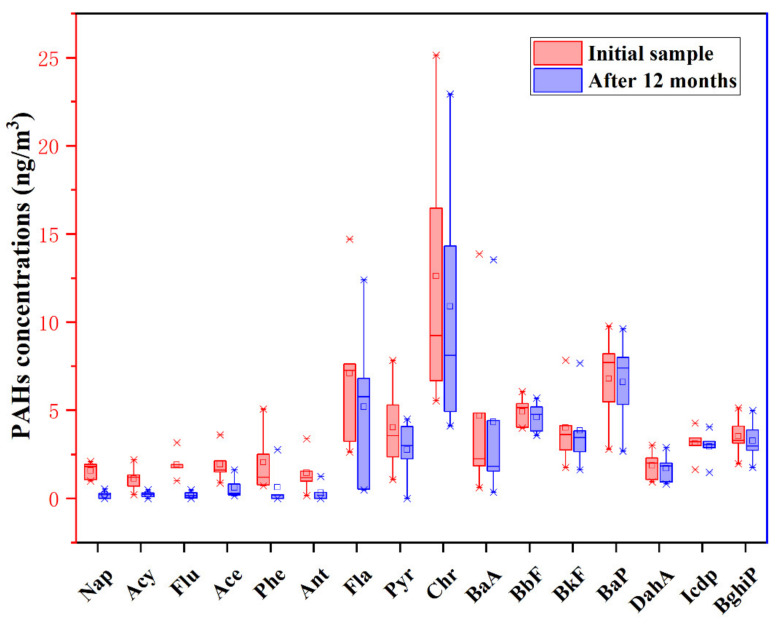
Concentration changes in 16 PAHs after storage for 12 months.

**Figure 5:**
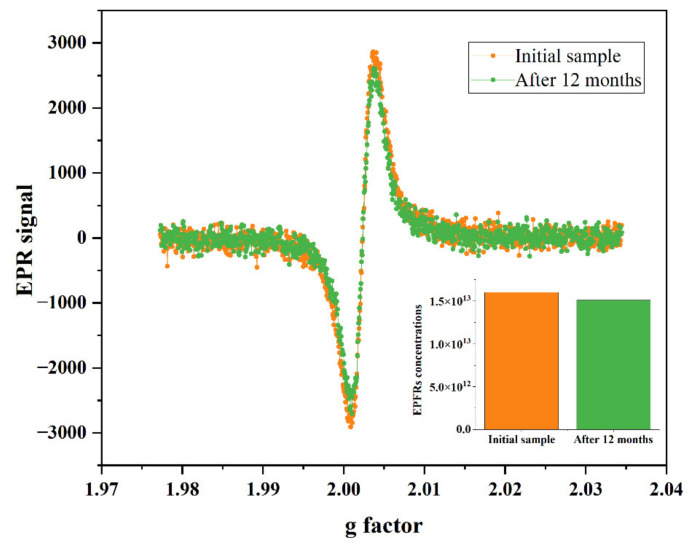
EPFR changes after storage for 12 months. The smaller graph shows EPFR concentration changes and the larger one shows the EPR signal change.

The EPFRs detected in atmospheric fine particles are often unpaired electrons formed during the degradation of organic compounds, such as PAHs. These are stabilized in the environment in conjugation with environmental media or through the delocalization of aromatic chemical bonds. To investigate whether there is an association between PAH degradation and EPFR formation, PAH concentrations (Figure 4) and EPR signal changes (Figure 5) during storage of PM_2.5_ samples were compared. The total concentration of PAHs in the original sample was 62.70 ng/m^3^, and after 12 months the total concentration decayed to 48.32 ng/m^3^. Figure 3 shows how the concentration of PAHs with low relative molecular masses decreased over the 12 months. This was attributed to the easier degradation and volatilization of these PAHs. However, under the same conditions, the EPR signal intensity and eigenvalues for the samples hardly changed. Studies have shown that the intermediate products formed by the degradation of PAHs are an important source of EPFRs in environmental media [36]. Moreover, some of the EPFRs will degrade under certain conditions during the generation process. Simultaneous generation and disassembly of EPFRs explains the weak changes in EPR signaling.

**Figure 6:**
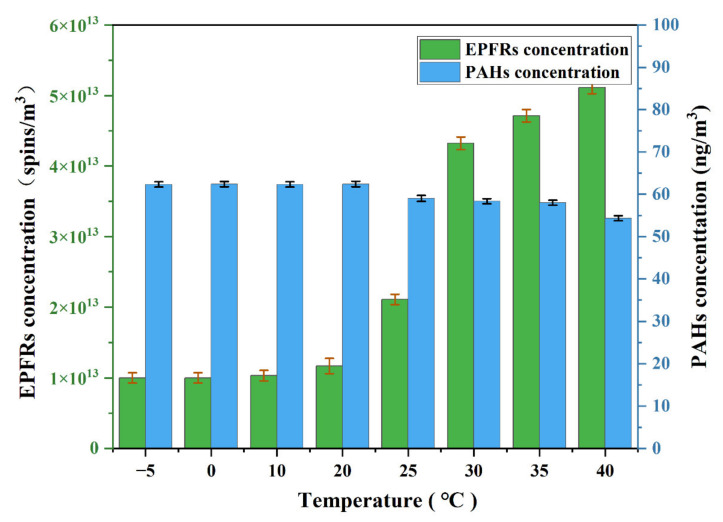
EPFR and PAH concentrations in PM_2.5_ samples at temperatures of −5–40 °C.

The correlation results in Figure 2 show that temperature has a strong correlation with EPFRs. Some studies have reported that aromatic molecules can form EPFRs at certain temperatures [37,38]. To explore the changes in EPFRs and PAHs in PM_2.5_ samples at different temperatures, the concentrations of PAHs and EPFRs at different temperatures were investigated. The PM_2.5_ samples were placed in the open system at different temperatures for 30 min, and then the concentrations of PAHs and EPFRs were measured rapidly. The results are shown in Figure 6. The concentrations of PAHs and EPFRs remained constant from −5 °C to 20 °C, which is because the two substances remain stable and do not transform into each other at low temperatures. At 25 to 40 °C, the concentration of PAHs decreased with the increasing temperature, and the concentration of EPFRs increased with the increasing temperature. This result is most likely explained by the conversion of PAHs to EPFRs. Some studies suggest that other factors may also have a slight effect on the generation of EPFRs [4,39]. Transition metals in PM_2.5_ catalyze the formation of EPFRs. In addition, the degradation process by which polyaromatic hydrocarbon structures decompose into aromatic subunits and form free radicals may contribute to the increase in the EPR signal [40].

### 3.5. Potential Exposure Risks of EPFRs

To date, standard principles have not yet been developed to assess the potential risk of exposure to EPFRs through PM_2.5_ inhalation in humans. Analogous organic radicals have been found in cigarette tar [41]. Consequently, the number of EPFRs equivalent to cigarette tar was used to evaluate the potential health risk of EPFRs. EPFR exposure was equivalent to 1.69–4.61 cigarette tar EPFRs per day in spring, 0.66–4.47 cigarette tar EPFRs per day in summer, 1.49–4.37 cigarette tar EPFRs per day in fall, and 2.26–8.40 cigarette tar EPFRs per day in winter. The exposure levels in winter were approximately two times higher than the levels recorded in the other three seasons. This may have been due to the considerable number of haze events that occurred in Shihezi that winter. High concentrations of PM_2.5_ lead to large numbers of EPFRs and greater human exposure.

**Figure 7:**
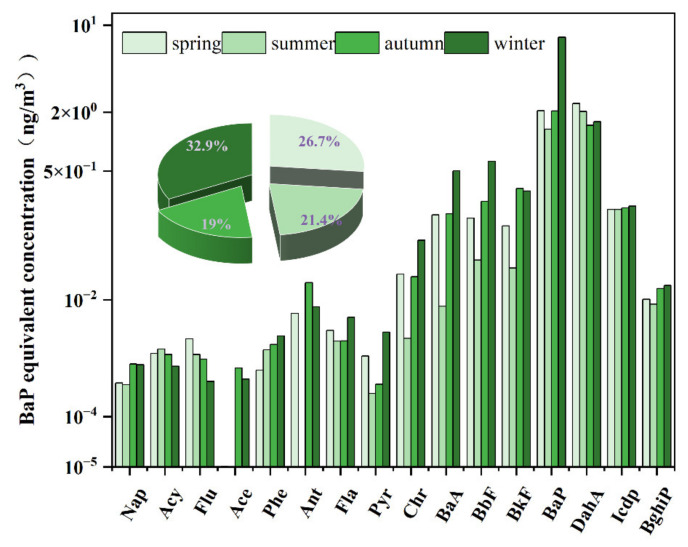
BaP_eq_ concentration from PAH inhalation exposure in four seasons.

The mean BaP_PAHs_ concentrations were highest in winter (18.12 ng/m^3^), followed by spring (14.74 ng/m^3^), summer (11.81 ng/m^3^), and fall (10.49 ng/m^3^), with an annual mean concentration of 13.79 ng/m^3^ (Figure 7). The equivalent numbers of cigarettes were used to represent the PAH exposure per person per day. PAH exposure was equivalent to 0.21 cigarette tar EPFRs per day in spring, 0.16 cigarette tar EPFRs per day in summer, 0.15 cigarette tar EPFRs per day in autumn, and 0.25 cigarette tar EPFRs per day in winter.

## 4. Conclusions and Study Implications

This study comprehensively determined the concentration levels and species characteristics of EPFRs in PM_2.5_, as well as the correlations between EPFRs and other influencing factors. The experimental results showed that there is a certain relationship between the degradation of PAHs and the generation of EPFRs. The number of EPFRs inhaled per person per day was equivalent to 0.66–8.40 cigarettes based on annual average concentrations in the atmosphere. EPFR exposure in Shihezi was about 15 times higher than that reported in the United States [41]. The results of our study indicate the daily concentrations of EPFRs in PM_2.5_ throughout the year and we compared the concentration levels for four seasons. In addition, we found a strong correlation between EPFRs and other conventional pollutants, indicating that there is a mutual conversion relationship between EPFRs and other pollutants. We focused on the relationship between the decay process in PAHs and the generation process in EPFRs and found that one source of secondary EPFRs in aerosols is the decomposition of PAHs. Shihezi’s EPFRs may mainly come from fossil fuels and vehicle exhaust emissions. Our results suggest that government measures to prevent pollution, such as reducing coal burning and limiting cars, may have a positive impact on reducing the concentrations of PAHs and EPFRs in the atmosphere.

This study presents a risk assessment for environmentally persistent free radicals in PM_2.5_ and the influence of air pollutants. It also provides alternative assessment methods to assess the potential health risks posed by PAHs and EPFRs in PM_2.5._ The effective assessment of EPFRs is significant for the protection of human health and safety and this study provides a methodology to evaluate the presence of a combination of pollutants in the atmosphere. The impacts of carbon emissions on human health have long been debated. This research is highly relevant for current scenarios. It can also provide a reference for future studies to determine health risks in highly contaminated areas. The implications of these findings can go a long way in assisting policy-makers to implement efforts to reduce carbon emissions for improved human health.

## Data Availability

Not applicable.

## Supplementary Materials

The following supporting information can be downloaded at: https://www.mdpi.com/article/10.3390/toxics10070341/s1, Figure S1: Seasonal distribution trends for 16 PAHs in PM_2.5_ in Shihezi between October 2020 and September 2021; Table S1: R regression equation, R2, detection limit, and mean recovery for 16 PAHs; Table S2: Toxicity equivalent factors.

## Abbreviations

EPR	Environmentally persistent radical
EPFR	Environmentally persistent free radical
HPLC	High-performance liquid chromatography
PAH	Polycyclic aromatic hydrocarbon
PM_2.5_	Fine particulate matter
