# Supplementary material for: Characteristics of Environmentally Persistent Free Radicals in PM2.5 and the Influence of Air Pollutants in Shihezi, Northwestern China
Source: Toxics. 2022 Jun 21;10(7):341. doi: 10.3390/toxics10070341 (PMC9321939; doi:10.3390/toxics10070341)
Supplement: Supplementary file 1 [file toxics-10-00341-s001.zip › toxics-1768949-SM.pdf]

toxics

MDPI

Supplementary Materials: Characteristics of Environmentally Persistent Free Radicals in PM2.5 and the Influence of Air Pollutants in Shihezi, Northwestern China

Feifei He, Jianjiang Lu , Zhuoying Li, Min Li, Zilong Liu and Yanbin Tong

Table S1. R regression equation, R^2, detection limit and mean recovery of 16 PAHs.

PAHs	Regression Equation	Correlation Coefficient R^2	Detection Limit（μg/L）	Average Recovery Rate
Nap	$Y = 5.04\times10^5X+9.83\times10^3$	0.9999	1.25	100.82%
Acy	$Y = 1.29\times10^5X+1.65\times10^4$	0.9994	5	98.63%
Flu	$Y = 5.70\times10^4X+2.33\times10^3$	0.9995	5.5	98.72%
Ace	$Y = 2.76\times10^5X+7.59\times10^3$	0.9998	4	95.19%
Phe	$Y = 8.05\times10^4X+1.02\times10^4$	0.9989	5.5	99.43%
Ant	$Y = 4.71\times10^4X+5.50\times10^3$	0.9996	10	99.11%
Fla	$Y = 1.14\times10^5X+6.72\times10^3$	0.9998	5	102.46%
Pyr	$Y = 5.52\times10^4X+3.43\times10^3$	0.9996	5	101.69%
Chr	$Y = 1.16\times10^5X+3.21\times10^3$	0.9999	5	92.99%
BaA	$Y = 1.26\times10^5X+4.60\times10^3$	0.9997	5	102.80%
BbF	$Y = 1.35\times10^5X+3.57\times10^2$	0.9996	5	103.88%
BkF	$Y = 9.25\times10^4X+8.01\times10^3$	0.9990	5	95.98%
BaP	$Y=7.84\times10^4X+4.71\times10^3$	0.9990	5	101.34%
DahA	$Y = 1.46\times10^5X+1.14\times10^4$	0.9996	5	87.17%
Icdp	$Y = 3.27\times10^4X+4.21\times10^3$	0.9993	5	106.45%
BghiP	$Y = 2.34\times10^5X+2.17\times10^3$	0.9995	5	100.05%

Table S2. Toxicity Equivalent Factors.

Compound	TEF_i	Compound	TEF_i
Nap	0.001	BaA	0.1
Acy	0.001	Chr	0.01
Ace	0.001	BbF	0.1
Flu	0.001	BkF	0.1
Phe	0.001	BaP	1
Ant	0.01	IcdP	0.1
Fla	0.001	DahA	1
Pyr	0.001	BghiP	0.01

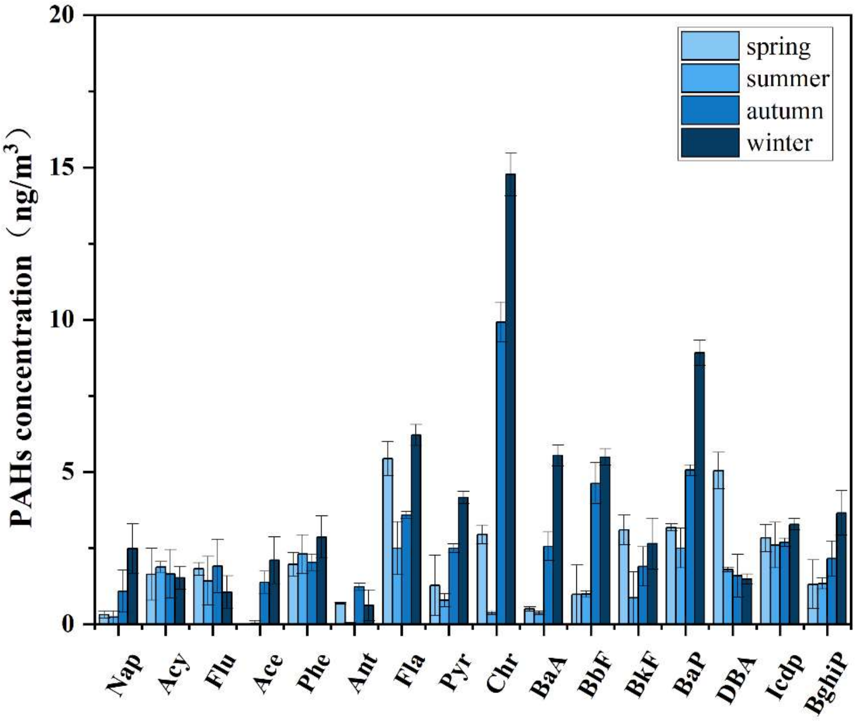

Figure S1. Seasonal distribution trends of 16 PAHs in $PM_{2.5}$ in Shihezi between October of 2020 and September of 2021.
